# Treatment Outcomes for Tuberculosis Infection and Disease Among Persons Deprived of Liberty, Uganda, 2020

**DOI:** 10.3201/eid3007.230611

**Published:** 2024-07

**Authors:** Deus Lukoye, Julius N. Kalamya, Anna Colletar Awor, Gail Gustavson, Joseph Kabanda, Odile Ferroussier-Davis, Charles Kajoba, Azaria Kanyamibwa, Leonard Marungu, Stavia Turyahabwe, Simon Muchuro, Lisa Mills, Emilio Dirlikov, Lisa J. Nelson

**Affiliations:** US Centers for Disease Control and Prevention, Kampala, Uganda (D. Lukoye, J.N. Kalamya, A.C. Awor, G. Gustavson, J. Kabanda, L. Mills, E. Dirlikov, L.J. Nelson);; Centers for Disease Control and Prevention, Atlanta, Georgia, USA (O. Ferroussier-Davis);; Uganda Prisons Service, Kampala (C. Kajoba, A. Kanyamibwa, L. Marungu);; Uganda Ministry of Health, Kampala (S. Turyahabwe, S. Muchuro)

**Keywords:** tuberculosis, TB, persons deprived of liberty, latent TB infection, TB preventive treatment, bacteria, respiratory infections, tuberculosis and other mycobacteria, Uganda

## Abstract

We report that unsuccessful treatment outcomes were 11.8% for tuberculosis (TB) disease and 21.8% for TB infection among persons deprived of liberty in Uganda Prisons Service facilities. Remedial efforts should include enhancing referral networks to ensure treatment continuity, strengthening data systems for complete outcome documentation, and prioritizing short-course treatment regimens.

Tuberculosis (TB) remains a major public health challenge and the most frequent cause of illness and death among persons living with HIV (PLHIV) (*1*). Globally, TB occurs in congregate settings marked by malnutrition, overcrowding, underlying illnesses or conditions such as HIV, and poor ventilation, which include correctional facilities (*2*–*4*). High TB prevalence in prisons contributes to illness and death not only among persons deprived of liberty (PDLs; e.g., persons who are incarcerated or otherwise being held in detention facilities) but also among the general population who interact with PDLs in prison or after a PDL’s release (*3*).

In 2020, the Uganda Prisons Service housed ≈65,000 PDLs across 259 facilities. The average stay was 19.1 months for capital offenders and 3.6 months for petty offenders. In Uganda, TB incidence was estimated to be 2,071/100,000 persons in prisons in 2019 (*5*). All PDLs are screened at entry into Uganda Prisons Service facilities for TB disease and HIV status to establish eligibility for TB preventive treatment (TPT). Case finding during incarceration is determined from symptom self-reporting. Since 2016, the treatment regimens recommended by the Uganda Ministry of Health have been isoniazid, rifampin, ethambutol, and pyrazinamide for 2 months, followed by isoniazid/rifampin for 2 months for TB disease and 6 months of isoniazid for TB infection (TBI). However, 2019–2021 Uganda Prisons Service data indicated suboptimal treatment outcomes for those regimens; the TB disease treatment success rate was 89% and cure rate was 65%, and TPT completion rate was 75% (*6*). To inform remedial interventions, we conducted a cross-sectional study to evaluate correlates of unsuccessful treatment outcomes for TB disease and TBI among PDLs in Uganda.

## The Study

In December 2021, we extracted data on PDLs who received TB disease or TBI treatment during January–December 2020 at 27 prisons that were selected according to availability of TB and HIV services at those sites (Appendix Figure). We abstracted sociodemographic and clinical information (age, sex, HIV status, incarceration stay, treatment initiation date, and outcome) from prison entry, TPT, and unit TB registers. TB disease data collected from unit TB registers were TB treatment history (new, previously treated), diagnosis (laboratory confirmed, clinical), and site (pulmonary, extrapulmonary) (*7*). We calculated the duration of prison stays before initiation of TB disease or TBI treatments. For TB disease treatment, we defined successful outcomes as cured or treatment completion (treatment was completed but no bacteriologic proof of cure) and unsuccessful outcomes as lost to follow-up (LTFU), death, treatment failure, or no documented outcome (*7*). For TBI treatment, we defined successful outcomes as treatment completed and unsuccessful outcomes as LTFU, death, or treatment stopped. When the outcome was treatment stopped, we collected data on reported reasons (*8*).

We calculated descriptive statistics and conducted bivariate analyses to determine associations between exposure and outcome variables. We used χ^2^ tests for categorical variables and *t*-tests for continuous variables to identify associations between independent and outcome variables. We considered variables with a p value of <0.05 and an estimate range within a 95% CI to be significant. We included all variables with a p value of <0.2 from bivariate analysis in the multivariate analysis. We used logistic regression to control for effect modification and confounders with dichotomous outcomes. We conducted analyses by using SAS version 9.4 (SAS Institute Inc., https://www.sas.com).

PDLs residing in the 27 selected prisons comprised 62.5% (38,005/60,771) of the total prison population in Uganda during December 2020; the median number of PDLs was 953 (range 79–5,998). During the study period, 1,117 PDLs were treated for TB disease; median age was 34 (interquartile range [IQR] 26–43) years, and most (1,098 [98.3%]) were men. The median number of TB patients per prison was 28 (IQR 6–68). Among the 1,116 with TB disease who had documented HIV status, 340 (30.5%) were HIV-positive. Only 7.3% (82/1,117) of PDLs were previously treated for TB disease (80 relapses and 2 returns after LTFU). Among the 985 (88.1%) PDLs who had successful outcomes, 446 (45.3%) were cured and 539 (54.7%) completed treatment. Among the 132 PDLs who had unsuccessful outcomes, 36 (27.2%) were LTFU, 35 (26.5%) died, 5 (3.7%) failed treatment, and 56 (42.4%) had no documented outcome (54 transferred out, 1 had multidrug resistant TB treatment, and 1 was not evaluated). The death rate among PDLs treated for TB disease was 3.1% (35/1,117). PDLs with laboratory-confirmed pulmonary TB (adjusted odds ratio [aOR] 2.2, 95% CI 1.03–4.81; p = 0.04) and extrapulmonary TB (aOR 1.7, 95% CI 1.11–2.54; p = 0.014) had higher odds of unsuccessful treatment outcomes than those with clinically diagnosed pulmonary TB (Table 1). PDLs whose prison stays were <6 months had higher odds of unsuccessful outcomes (aOR 3.1, 95% CI 1.60–6.11; p = 0.0008) than those who stayed >6 months.

**Table 1:** Characteristics and treatment outcomes among persons deprived of liberty who had TB disease in Uganda prisons, January–December 2020*

Variable	Total, no. (%)	Successful treatment, no. (%)	Unsuccessful treatment, no. (%)	Crude OR (95% CI)	p value	aOR (95% CI)	p value
Patient sex							
F	19 (1.7)	18 (94.7)	1 (5.2)	0.4 (0.06–3.12)	0.39	0.5 (0.07–3.88)	0.51
M	1,098 (98.3)	967 (88.2)	131 (11.8)	Referent		Referent	
Age, y							
<35	858 (76.8)	784 (91.4)	74 (8.6)	1.2 (0.83–1.73)	0.34	1.1 (0.76–1.63)	0.58
>35	259 (23.2)	202 (78.0)	57 (22.0)	Referent		Referent	
Patient category							
New	1,035 (92.7)	912 (88.1)	123 (11.9)	1.2 (0.57–2.58)	0.61	1.5 (0.66–3.54)	0.32
Previously treated	82 (7.3)	74 (90.2)	8 (9.8)	Referent		Referent	
TB form							
Pulmonary, laboratory confirmed†	631 (56.5)	548 (86.8)	83 (13.2)	1.6 (1.06–2.40)	0.03	2.2 (1.03–4.81)	0.04
Extrapulmonary	56 (5.0)	46 (82.1)	10 (17.9)	2.3 (1.07–4.92)	0.02	1.7 (1.11–2.54)	0.014
Pulmonary, clinical diagnosis	428 (38.5)	391 (91.7)	37 (8.6)	Referent		Referent	
HIV status, n = 1,116‡							
Negative	776 (69.7)	683 (88.0)	93 (11.9)	0.9 (0.62–1.39)	0.72	0.9 (0.57–1.46)	0.703
Positive	338 (30.3)	300 (88.8)	38 (11.2)	Referent		Referent	
Length of incarceration before treatment							
<6 mo	908 (81.4)	787 (86.7)	121 (13.3)	3.0 (1.58–5.91)	0.0010	3.1 (1.60–6.11)	0.0008
>6 mo	208 (18.6)	198 (95.2)	10 (4.8)	Referent		Referent	

*aOR, adjusted odds ratio; TB, tuberculosis. †Pulmonary TB was confirmed by bacteriologic laboratory tests. ‡Data are for a total of 1,116 patients with TB disease who had documented HIV status.

A total of 2,672 PDLs were treated for TBI; the median age was 30 (IQR 26–38) years, and most of those treated (2,337 [87.5%]) were men. Of 2,668 PDLs with documented HIV status, 2,468 (92.5%) were HIV-positive. The median number of PDLs treated for TBI per prison was 70 (IQR 44–126). Overall, 79.1% (2,113/2,672) completed TBI treatment. Among 555 PDLs who had unsuccessful outcomes, 245 (44.1%) transferred out, 207 (37.3%) were LTFU, 98 (17.7%) stopped treatment (31 experienced adverse events, 11 had TB disease and appropriate treatment was started, and 56 did not have reported reasons), 5 (0.9%) died (including 4 PLHIVs), and 4 had missing data. The death rate among PDLs treated for TBI was 0.2% (5/2,672). Higher risk for unsuccessful TBI treatment outcomes was associated with PDLs who stayed <6 months (aOR 1.8, 95% CI 1.28–2.48; p = 0.0006), were men (aOR 1.60, 95% CI 1.10–2.27; p = 0.014), and were PLHIVs (aOR 2.13, 95% CI 1.42–3.21; p = 0.0003) (Table 2).

**Table 2:** Characteristics and treatment outcomes among persons deprived of liberty who had TB infections in Uganda prisons, January–December 2020*

Variable	Total, no. (%)	Successful treatment, no. (%)	Unsuccessful treatment, no. (%)	Crude OR (95% CI)	p value	aOR (95% CI)	p value
Patient sex							
M	2,337 (87.5)	1,829 (78.2)	508 (21.8)	1.7 (1.23–2.36)	0.0012	1.6 (1.10–2.27)	0.014
F	335 (12.5)	288 (86.0)	47 (14.0)	Referent		Referent	
Age, y							
<35	1,720 (64.5)	1,359 (79.0)	361 (21.0)	1.0 (0.85–1.26)	0.7495	1.1 (0.88–1.37)	0.44
>35	948 (35.5)	754 (79.5)	194 (20.5)	Referent		Referent	
HIV status							
Negative	200 (7.5)	166 (83.0)	34 (17.0)	Referent		Referent	
Positive	2,468 (92.5)	1,947 (78.9)	521 (21.1)	1.3 (0.89–1.91)	0.1684	2.1 (1.42–3.21)	0.00
Length of incarceration before treatment							
<6 mo	2,212 (84.6)	1,714 (77.5)	498 (22.5)	1.8 (1.34–2.43)	<0.0001	1.8 (1.28–2.48)	0.0006
>6 mo	403 (15.4)	347 (86.1)	56 (13.9)	Referent		Referent	

*aOR, adjusted odds ratio; TB, tuberculosis.

TB is common in Uganda Prisons Service facilities and, despite improvements among the general population, treatment outcomes among PDLs remain suboptimal (*6*). Renewed efforts are needed to improve treatment outcomes, especially for PDLs who have extrapulmonary infection or laboratory-confirmed pulmonary TB or who are PLHIVs (*9*,*10*). Because of transmission risks within this congregate setting, TPT could be further expanded, including among HIV-negative persons. Unsuccessful outcomes did not differ markedly according to HIV status among patients with TB disease. Reported deaths among PLHIV treated for TBI might be attributed to opportunistic infections or missed TB disease diagnoses (*11*).

Short prison stays are challenging for successful TB treatment (*12*). Enhanced referral networks could ensure treatment continuity, including during prison transfers and after release. Strengthened data systems are needed to properly document and report treatment outcomes regardless of release or transfer status. Finally, World Health Organization–recommended short-course treatment regimens for TB disease and TBI could be prioritized for prisons to improve treatment completion (*13*,*14*) and documentation, given the frequent transfers, short average stay, and suboptimal continuity of care after release.

The first limitation of our study is that we could not evaluate outcomes among PDLs who transferred out or those without documented outcomes, which could negatively skew results. Second, data limitations precluded analysis of other known risk factors for unsuccessful treatment outcomes (e.g., homelessness, substance abuse, previous imprisonment, presence of cavitary disease) (*15*). Finally, the concurrent COVID-19 outbreak during the study period, which disturbed health service provisions, could have affected treatment outcomes, limiting generalizability of findings to the years before and after the outbreak.

## Conclusions

We found suboptimal treatment outcomes for PDLs with TB disease and TBI in Uganda Prisons Service facilities. Remedial efforts are needed to enhance referral networks to ensure treatment continuity, strengthen data systems for complete outcome documentation, and prioritize short-course treatment regimens.

Appendix Additional information for treatment outcomes for tuberculosis infection and disease among persons deprived of liberty, Uganda, 2020.
