# Supplementary material for: Treatment Outcomes for Tuberculosis Infection and Disease Among Persons Deprived of Liberty, Uganda, 2020
Source: Emerg Infect Dis. 2024 Jul;30(7):1402–5. doi: 10.3201/eid3007.230611 (PMC11210659; doi:10.3201/eid3007.230611)
Supplement: Appendix — Additional information for treatment outcomes for tuberculosis infection and disease among persons deprived of liberty, Uganda, 2020. [file 23-0611-Techapp-s1.pdf]

Article DOI: https://doi.org/10.3201/eid3007.230611
EID cannot ensure accessibility for supplementary materials supplied by authors. Readers who have difficulty accessing supplementary content should contact the authors for assistance.

Treatment Outcomes for Tuberculosis Infection and Disease Among Persons Deprived of Liberty, Uganda, 2020

Appendix

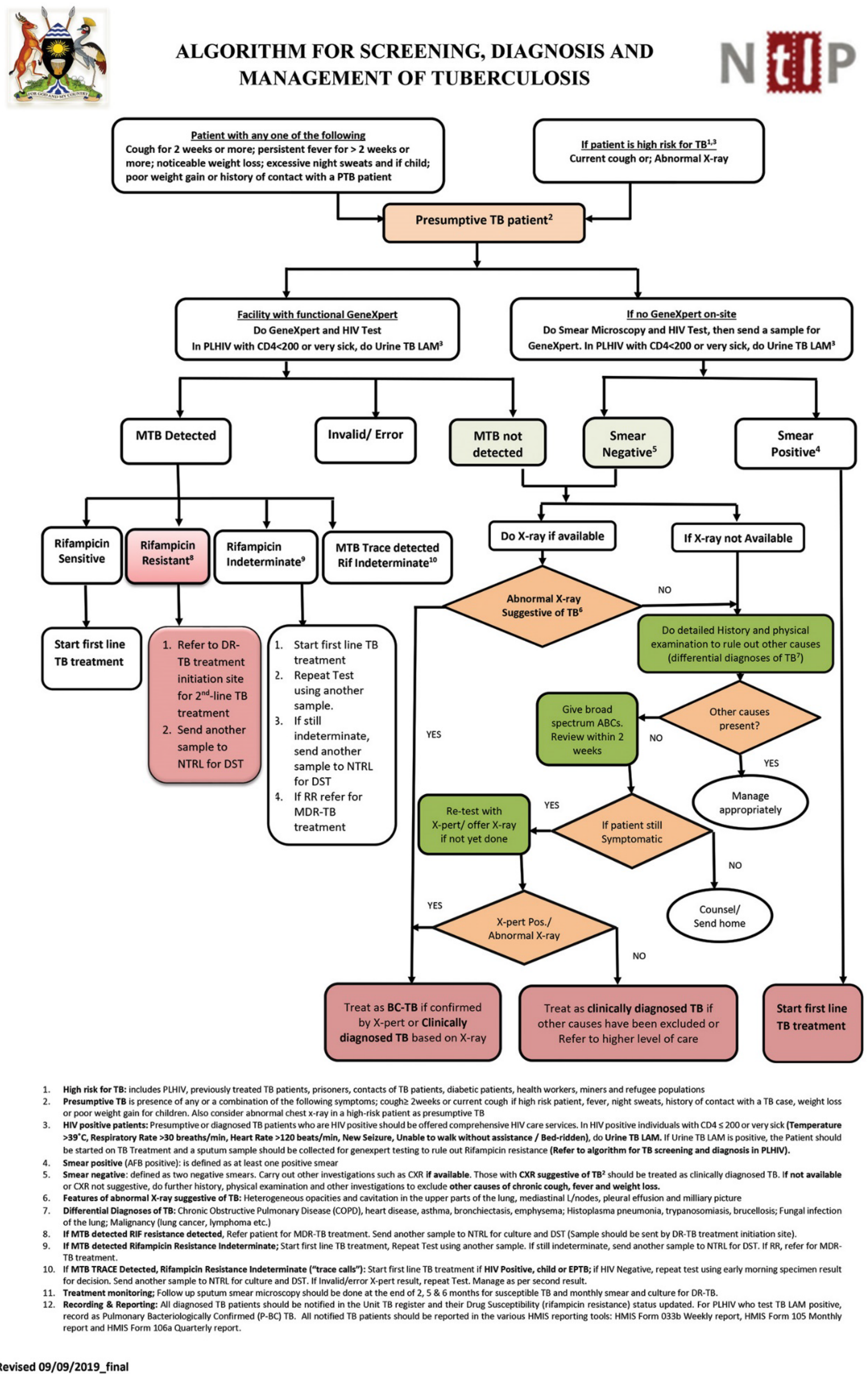

Appendix Figure. Algorithm used in Uganda for screening, diagnosis, and management of tuberculosis.
